# Democratic librarianship: the role of the medical library in promoting democracy and social justice

**DOI:** 10.5195/jmla.2020.852

**Published:** 2020-01-01

**Authors:** Elaine Russo Martin

**Affiliations:** Director of Library Services, Countway Library, Harvard Medical School, Boston, MA, elaine_martin@hms.harvard.edu

## Abstract

Evidence suggests that Erich Meyerhoff was one of the first practitioners of democratic librarianship throughout his long and productive life. This essay defines democratic librarianship in the context of democratic ideals and social justice and posits actions that the profession should be taking to thrive and lead in a multicultural environment, including being a place for active engagement, crucial conversations, and debate. Democratic librarianship is broader than social justice but incorporates social justice ideals in promoting a socially just and democratic society.

Libraries…are essential to the functioning of a democratic society;…and libraries are the great tools of scholarship, the great repositories of culture, and the great symbols of the freedom of the mind. [1]

—Franklin D. Roosevelt

## INTRODUCTION

How do we define democracy? The most commonly understood definitions of democracy combine the ideas of information, education, equity, and social justice: “Democracy is, in essence, the concept of social fairness and justice” [2]. Libraries serve society, and academic medical libraries specifically serve society by improving human health through equal access to quality health information no matter the person’s social status, sexual orientation, socioeconomic status, racial or ethnic identity, or disability status. Democracy also depends on an educated and informed electorate:

An informed public constitutes the very foundation of a democracy; after all, democracies are about discourse—discourse among the people. If a free society is to survive, it must ensure the preservation of its records and provide free and open access to this information to all its citizens. It must ensure that citizens have the skills necessary to participate in the democratic process. It must allow unfettered dialogue and guarantee freedom of expression. All of this is done in our libraries, the cornerstone of democracy in our communities. [3]

Democracy embodies both values and professionalism. These values include respect for: (1) diversity and inclusion, (2) every human being, (3) equal access to evidence-based information, (4) the truth, (5) lifelong learning, and (6) courageous and socially responsible action in professional practice. The definitions of democracy and its accompanying values capture the essence of what we do in our academic medical libraries. Professionalism includes democratic ideals such as: (1) providing accurate evidence-based health information to all who need it; (2) educating our students and faculty through information literacy programs about information retrieval, access, and evaluation; and (3) having our spaces be the center of community engagement and social justice activities on our campuses.

Libraries are defenders of human and social rights through providing services and questioning the ideology of library neutrality. Medical libraries, in particular, focus on human rights and principles of social justice by, for example, supporting open access repositories, highlighting collections and publications from women and other minority physicians, and opening their spaces for difficult conversations and debate.

The purpose of this essay is to discuss democratic medical librarianship and the role of the health sciences library in supporting a socially just and democratic society. Why write about the role of the medical library in democracy? We live and work in a time of crisis of democracy. The democratic values we have held dear are being questioned with the rise of white supremacy and white nationalism, and the breakdown of long-held treaties with allies, along with verbal and Twitter attacks on truth, science, and the news media, to name just a few.

A culture of policy making via Twitter rants and the passage of state laws by extremists are threatening the rights of the underserved, immigrants, ethnic and racial minorities, and women. The integrity of scholarship, freedom of expression and thought, our institutions of higher education, and even the survival of US democratic elections are under attack. Under the guise of protecting the public’s health, new state and local laws overturning access to health care, health privacy, and bodily health choices have been passed. Health sciences libraries are part of the solution. We are living in a world of “post-truth politics,” where “fake news” and “alternative facts” compete with evidence-based, peer-reviewed, expert, and scientifically proven information [4].

While “post-truth politics” is not new, the proliferation of social media and Internet sites where everyone and anyone can publish and post emotionally charged commentary as fact and then make that information instantaneously available all over the globe makes our response more urgent. Now more than ever, medical librarians need the courage to actively affirm our values and professional expertise in a democratic and socially just society and take our professional place in our institutions.

## DEMOCRATIC LIBRARIANSHIP

Expressing and acting on our roles in democracy and social justice can be termed democratic librarianship. Democratic librarianship is an expression of the democratic (“with a small d”) values we hold dear: to see users as individuals, to really listen and hear their stories with sincerity and concern, to recognize the worth and dignity of every person who comes into our libraries, and to have the courage to uncover the truth [5]. It is the actions we take to move health information from ignorance to the truth through accessibility and equity of evidence-based health information from the ivory tower to the public realm. It is extending the social justice mindset of our libraries to our reference, collections, archives, and special collections services. It encompasses a commitment to diversity and inclusion within and outside our libraries to the communities in which we work and collections that we house: “By collecting, accessing, organizing, preserving, and disseminating the rich diversity of human expression in all its varied forms, libraries ensure free speech, self-government, and individual enrichment” [6].

In the world in which we live today, democratic librarianship also encompasses the act of questioning the myth of library neutrality. Our buying decisions, our cataloging practices, and our use of commercial search engines, for example, are not neutral. A neutral stance would not care about human rights violations or social and economic inequalities [7]:

The ideology of political neutrality, unfortunately keeps professionals such as journalists, teachers, and librarians—as well as citizens—from understanding the relationship between power and the professions. Any claims to such neutrality is illusory; there is no neutral ground on which to stand anywhere in the world. [8]

Continuing to resist taking a stand on important issues related to preserving our democracy threatens our professional relevance. Providing accurate, evidence-based health information about the measles vaccine or HIV/AIDS is not neutral. Medical librarians cannot be neutral and be trusted advocates for improving the health of the communities they serve.

Therefore, it is time for medical librarians to move from passivity to activism with respect to addressing the injustices in health care [9]. It is our professional responsibility to go beyond recognizing the health information and health care needs of the underserved and disadvantaged and work toward improving their health. Medical librarians do so by actively engaging in the civic life of the communities we serve. In exercising our civic duty and professional responsibilities, medical librarians must move beyond creating awareness and informing to taking empathetic action, working toward shaping public policy, and participating in the national debate on these issues. It is our professional responsibility to become civic partners with those who address the health care needs of the underserved (such as the LGBT community) and the disadvantaged (i.e., the poor) and actively participate in improving their health status. It is this commitment to advancing everyone’s health through providing access to quality health information, offering health education, working to support human rights, creating opportunities for community engagement, fostering social justice, and participating in civic activism that cements our professional role in a democratic society:

Libraries, whether they are public or special, academic or school, are a cornerstone of the communities they serve and are essential to the preservation of rights. Libraries provide the ideas, resources, and information imperative for education, work, recreation, and self-government. [10]

Trust in authority and professional practice is increasingly fragile. Practicing democratic librarianship is one way for medical librarians to express the values we hold dear as a profession, demonstrate these values in our everyday work, and build on our reputation as a trusted profession. Professionalism and expertise play a crucial role in a democracy. Medical librarians must develop a new professional orientation that shifts the focus away from the library as a warehouse for books and journals, and away from the emphasis on the latest technological products to a consciousness that puts the users of information front and center.

Medical librarians, while still valuing the specialized knowledge of their profession, can be more democratic by engaging their users in the decision-making process, listening to their needs, and working collaboratively with them on providing library policies and services that affect them [7]. Democratic librarianship is participatory and respectful. It is bridge building. Through their professional expertise, practitioners of democratic librarianship advocate for, connect to, and engage with local communities in using library spaces. In addition, they work in tandem with other people and institutions to develop library policies and solve information problems. By adopting the practices and principals of democratic librarianship, medical librarians can rebuild the trust between professional expertise and the public that is under attack today [11].

## ERICH MEYERHOFF AND DEMOCRACY AND SOCIAL JUSTICE

I did not know Erich Meyerhoff. But everything I have read about him and heard from others has convinced me that Erich was an early practitioner of democratic librarianship and social justice. His Janet Doe Lecture, his obituary, and his professional colleagues’ remembrances of him, as well as his own personal history and courage during World War II (WWII), give us insights into his belief in a larger role for our profession beyond a curatorial function to that of service to society. His early advocacy for equal access to health information and for the direct role of medical librarians in improving society’s health; his commitment to the truth; his laser-like focus on diversity, especially in the advancement of women and support of international librarianship; and his active participation in MLA’s legislative agenda echo today’s calls for a more activist medical librarian.

Erich wrote of our expanded professional values and purpose in his 1977 Janet Doe Lecture, when he said:

We are on the way to a new self-consciousness and to a better understanding of our purpose and function…These are clearly in the area of communication and the transfer of knowledge to researchers, teachers, students, the sick and the healthy. [12]

His mention of the sick as a core user population for our professional practice was novel for the time. Previous Doe lecturers had focused on defining our values based on what we do or tasks to be performed. Erich was one of the few early lecturers who defined our values and purpose in terms of whom we serve and our impact on the public’s health.

In this same lecture, Erich introduced the topic of evidenced-based librarianship, though that term did not come into fashion until much later. His discussion regarding the recognition of the medical librarian’s unique contribution to the knowledgebase (one of the elements that defines a profession) focused on the librarian’s emphasis on accuracy in bibliographic technique. The elimination of error, the need for the careful establishment of the authenticity of documents, and the objective evaluation or “weighing of the evidence” is a mandate for medical librarians in order to carefully and *truthfully record significant historical events* and is a cornerstone to our professional practice [12].

Erich is most known in the professional literature as the founding director (1961–1967) of the Medical Library Center of New York. His vision was that the center would house a set of shared bibliographic resources (mainly periodicals) and services to provide research and experimentation in advancing medical librarianship and medicine:

The Center operated on a cooperative, collaborative basis as a “library for other libraries.” Its main purpose, initially, was to be a central institution for housing and distributing journals because of the enormous growth of the journal literature and inadequate library storage space in the major area medical schools for rarely used materials. [13]

The Medical Library Center of New York was an early adaptation of our modern day shared physical and digital repositories. It operated on the principles of open, inclusive, and equitable scholarly communications including open access and open data and what we now call the “The FAIR guiding principles for scientific data management and stewardship” (making data findable, accessible, interoperable, reusable) [14]. For example, the computer source code for the center’s union catalog was available to everybody who wanted it [13].

Following on Erich’s commitment to open, inclusive, and equitable access to the world’s scientific evidence-based research literature, he naturally focused his energies on where the profession of medical librarianship falls short: that of diversity, inclusion, and social justice. Those who worked for him and with him emphasized his commitment to mentorship: growing junior librarians and recruiting and hiring what one colleague called “a rainbow of races and nationalities. Erich liked to give people a chance and practiced this belief as director” [15]. He recognized the value of human differences and the worth and dignity of all people. He advocated for the Medical Library Association to become an international association, to not only include members outside the United States, but also for the *Bulletin of the Medical Library Association* (former name of the *Journal of the Medical Library Association [JMLA]*) to include research papers that transmit the interests of health sciences librarianship throughout the world [16].

Erich was a champion of women’s rights. He ended his Janet Doe Lecture stating that “Secondly, as members of a profession composed overwhelmingly of women we will gain from the drive for equality and recognition of excellence which will continue to affect our political and social life in the foreseeable future” [12]. Erich also understood that diversity and inclusion go beyond gender to race, language, socioeconomic status, social class, and sexual orientation. He promoted a sense of belonging that recognized the talents and backgrounds of the many as valuable and promoted the intersection of social categories and their valuable impact on scholarly research. Ellen Gay Detlefsen described her first meeting with him:

I first met Erich in 1970 when he, together with Eric Moon (then the editor of *Library Journal*) organized a special meeting of the Archons of Colophon, a society of leading library administrators (all men!) in New York City. The meeting was to address the issues of emerging social concerns in librarianship at a time when the country was in the throes of the many movements for change; Erich asked me to speak on women’s issues, together with E.J. Josey on black civil rights and Israel Fishman on the emerging gay rights movement. We three did, and Erich took great delight in telling me that this was the first time that a woman had ever spoken (or even attended!) an Archons meeting. He was our champion for social justice! [15]

Erich understood the importance of diversity of voices and the value of an engaged community from multiple organizations and collaborations that are needed for the pursuit of excellence and truth. Erich believed in an individual as well as collective responsibility for social change as evidenced in his own personal record of service. For Erich, “Citizenship is not a spectator sport” [17].

Erich demonstrated courage in his professional and personal life because he was one of the Ritchie Boys. The Ritchie Boys consisted of approximately 15,200 servicemen who were trained for US Army Intelligence during WWII at the secret Camp Ritchie training facility outside of Maryland. Some of them were young Jewish refugees like Erich, born in Germany and recruited because of their fluency in German. They had been drafted into or volunteered to join the US Army and served as important counterintelligence agents during WWII and as translators during the Nuremberg trials [18, 19].

## CONCLUSION

The value of the medical library for a functioning democracy goes beyond its original purpose and function as a warehouse for print books, journals, and other physical holdings. Erich Meyerhoff understood this. The medical library must move beyond its traditional role as a transaction unit (i.e., checking out books and providing links to online journal articles) to more relational interactions that turn outward into the communities they serve. One way to accomplish this is for the library to become a place for developing relationships amongst a variety of people from multiple disciplines, countries, races, and backgrounds. The library can be a place for active civic engagement and its community spaces can be used for crucial conversations and debate. Medical librarians bring diverse communities together from a variety of subject disciplines and specialties as well as the general public to exchange information, ask questions, research information, evaluate what they find, and learn about and cooperatively solve problems of common concern. Libraries are places for coalitions, partnerships, and active citizenship to occur.

Ninth Librarian of Congress Archibald MacLeish said, “Librarians must become active not passive agents of the democratic process” [20]. Medical libraries must not only educate and inform, but also engage with their users. Medical librarians are poised to lead this cause, create civic spaces, and engage their students, their faculty, and the public in social justice curricular activities. They need to reclaim their mission of improving the public’s health by using their spaces to encourage diverse students and faculty to gather with the public together in safe spaces or commons areas where interprofessional, nonconfrontational, deliberative discussion, and action can occur to find ways to improve the nation’s health. In this way, the medical library can become the intellectual and spiritual heart of the entire academic institution.

If medical libraries are to fulfill their civic and health mission in the information age, medical librarians must become activist librarians and practice democratic librarianship [9]. Such collective professional action will result in a revitalized role for the academic medical librarian as “the connective tissue that binds the community together” and result in “better communities and improved lives” [21]. Democratic and social justice librarianship needs to be central to the mission and purpose of medical librarianship and a core value of the profession. We must develop a professional commitment to addressing the issues of society with respect to access to evidence-based health information and use our information resources, spaces, and expertise to solve the relevant societal issues of today. This takes courage, a dedication to the truth, and a commitment to diversity and inclusion. Erich Meyerhoff’s career embodied these principles. The future of our democracy depends on the rest of us doing so, too.

## 

**Elaine Russo Martin, FMLA,** elaine_martin@hms.harvard.edu, Director of Library Services, Countway Library, Harvard Medical School, Boston, MA
